# BUB1/KIF14 complex promotes anaplastic thyroid carcinoma progression by inducing chromosome instability

**DOI:** 10.1111/jcmm.18182

**Published:** 2024-03-18

**Authors:** Tiefeng Jin, Lingling Ding, Jinming Chen, Xiaozhou Zou, Tong Xu, Zixue Xuan, Shanshan Wang, Jianqiang Chen, Wei Wang, Chaozhuang Zhu, Yiwen Zhang, Ping Huang, Zongfu Pan, Minghua Ge

**Affiliations:** ^1^ Otolaryngology & Head and Neck Center, Cancer Center, Department of Head and Neck Surgery Zhejiang Provincial People's Hospital, Affiliated People's Hospital, Hangzhou Medical College Hangzhou China; ^2^ Center for Clinical Pharmacy, Cancer Center, Department of Pharmacy Zhejiang Provincial People's Hospital, Affiliated People's Hospital, Hangzhou Medical College Hangzhou China; ^3^ Department of Pathology, Laboratory Medicine Center Zhejiang Provincial People's Hospital, Affiliated People's Hospital, Hangzhou Medical College Hangzhou China; ^4^ Key Laboratory of Endocrine Gland Diseases of Zhejiang Province Hangzhou China; ^5^ Clinical Research Center for Cancer of Zhejiang Province Hangzhou China

**Keywords:** anaplastic thyroid carcinoma, BUB1 mitotic checkpoint serine/threonine kinase, chromosome instability, phosphorylation, proliferation

## Abstract

Chromosome instability (CIN) is a common contributor driving the formation and progression of anaplastic thyroid cancer (ATC), but its mechanism remains unclear. The BUB1 mitotic checkpoint serine/threonine kinase (BUB1) is responsible for the alignment of mitotic chromosomes, which has not been thoroughly studied in ATC. Our research demonstrated that BUB1 was remarkably upregulated and closely related to worse progression‐free survival. Knockdown of BUB1 attenuated cell viability, invasion, migration and induced cell cycle arrests, whereas overexpression of BUB1 promoted the cell cycle progression of papillary thyroid cancer cells. BUB1 knockdown remarkably repressed tumour growth and tumour formation of nude mice with ATC xenografts and suppressed tumour metastasis in a zebrafish xenograft model. Inhibition of BUB1 by its inhibitor BAY‐1816032 also exhibited considerable anti‐tumour activity. Further studies showed that enforced expression of BUB1 evoked CIN in ATC cells. BUB1 induced CIN through phosphorylation of KIF14 at serine1292 (Ser^1292^). Overexpression of the KIF14^ΔSer1292^ mutant was unable to facilitate the aggressiveness of ATC cells when compared with that of the wild type. Collectively, these findings demonstrate that the BUB1/KIF14 complex drives the aggressiveness of ATC by inducing CIN.

## 1 INTRODUCTION

Recently, the incidence of thyroid carcinoma (TC) has been on a sharp rise, making it the fourth most frequent malignancy in the world.^1^ Papillary thyroid carcinoma (PTC) remains the most frequent TC subtype, accounting for nearly 85%, and most of which are curable.^2^ Whereas, anaplastic thyroid carcinoma (ATC) is a rare but highly malignant TC subtype, which approaches half of the deaths from TC. Unlike other TC types, it grows quickly with highly invasive behaviours, with over 70% of which undergoing lymph node metastasis and extrathyroidal extension, and 40% suffer distant metastasis.^3^ It is reported that the median survival period of ATC patients was only 3 to 7 months, and there is no effective treatment that could prolong the overall survival of patients.^4^, ^5^ Therefore, it is imperative to uncover the molecular mechanism behind ATC.

The high‐throughput sequencing of clinical tumour samples indicated enormous individual intra‐tumour genetic heterogeneity.^6^ CIN is recognized as one of the primary drivers of tumour heterogeneity and clonal diversification, which provides genetic diversity leading to cancer progression.^7^ It has been reported that CIN promotes metastatic dissemination and leads to poor prognoses and drug resistance.^8^, ^9^, ^10^, ^11^ In CIN, there is an intercellular difference in chromosome number or structure resulting from partial or whole chromosome loss or gain, and frequently forms aneuploidy of cellular chromosomes.^12^, ^13^ Previous studies have demonstrated that CIN represented a significant contributor to TC progression since chromosome abnormalities elevate from differentiated thyroid carcinoma (DTC) to poorly differentiated thyroid carcinoma and ATC.^14^, ^15^, ^16^, ^17^ Consequently, overexpression of genes associated with CIN is one of the hallmarks of ATC.^18^ Hence, it is fundamentally essential to identify the mechanism of CIN underlying ATC progression.

Spindle assembly checkpoint (SAC) abnormality is the potential cause accounting for CIN and has attracted more interest in the past few years.^19^ During mitosis and meiosis, the SAC prevents incorrect chromosome segregation from maintaining genome stability.^20^ BUB1 mitotic checkpoint serine/threonine kinase (BUB1) has been reported to make a central contribution to the mitotic chromosome alignment and the SAC.^21^ Thus, chromosome segregation defects have been observed in nonmalignant cells with BUB1 inactivation.^22^ Although BUB1 overexpression is a common phenomenon related to tumour proliferation in various solid tumours, studies of BUB1 in tumour CIN have been limited.^23^, ^24^, ^25^, ^26^ Our previous study demonstrated that BUB1 acts as the critical kinase in the protein–protein interaction network of ATC.^27^ Wada et al. also revealed that BUB1 expression was higher in tumours than in adenoma or normal tissue, and ATC expression was significantly higher than DTC expression.^28^ However, the biological function of BUB1 and its downstream signal in mediating CIN has not been well recognized in ATC.

In this work, we revealed that ATC overexpressing BUB1 is associated with worse outcomes. Antitumor activity of ATC proliferation, cell cycle arrest, invasion, migration and tumorigenesis were observed when BUB1 was inhibited. We demonstrated that BUB1 significantly increased CIN through the phosphorylation of KIF14 at Ser^1292^. Taken together, these results provide vital views on the function of BUB1/KIF14 complex signalling as a promising therapeutic target for ATC.

## 2 MATERIALS AND METHODS

### 2.1 Bioinformatics analysis

Gene Expression Profiling Interactive Analysis (GEPIA, http://gepia.cancer‐pku.cn) was retrieved to obtain BUB1 expression data.^29^ In addition, it was also utilized to examine BUB1 stage plots based on individual cancer stages and an expression correlation of BUB1 and KIF14. The online GEO2R tool (http://www.ncbi.nlm.nih.gov/geo/geo2r) was employed to investigate BUB1 and KIF14 expression among different TC subtypes in microarray datasets GSE33630 (49 PTC, 11 ATC, and 45 normal tissue (NT) samples), GSE29265 (20 PTC, 9 ATC and 20 NT samples) and GSE65144 (13 NT and 12 ATC samples).^30^, ^31^, ^32^ Based on the Kaplan–Meier plotter (https://kmplot.com), survival analyses of BUB1 and KIF14 were performed in TC patients.^33^ The median values were set as the cutoff value. Gene Set Enrichment Analysis (GSEA) was performed to investigate BUB1‐related chromosome signal in ATC to classify biological terms and conducted enrichment analysis in R (version *R4.0.4*) by a clusterProfiler R package (version *3.18.0*). Pathway enrichment analysis of the Kyoto Encyclopedia of Genes and Genomes (KEGG) was generated via GSEA based on BUB1 expression.

### 2.2 Patients and tissue samples

All research was approved by the Ethics Committee of Zhejiang Provincial People's Hospital. We retrospectively collected PTC samples from patients, who had undergone one‐stage surgery from January 2006 to January 2010. The correlation between BUB1 and PFS was examined on 232 PTC specimens made into tissue microarrays. Table 1 lists the basic clinicopathological information. We also collected non‐tumorous tissues, PTCs and ATCs to analyse the expression of BUB1.

**TABLE 1:** Clinicopathological features of 232 patients with thyroid cancer.

Variables	Stratification	BUB1 expression		*p*
		Low (*n* = 123)	High (*n* = 109)	
Age (years)	<55	100	95	0.224
	≥55	23	14	
Gender	Men	28	30	0.403
	Women	95	79	
Bilaterality	Unilateral	98	86	0.884
	Bilateral	25	23	
Tumour number	Solitary	88	72	0.367
	Multiple	35	37	
Maximal tumour diameter	<1 cm	23	27	**0.047** *
	1–4 cm	87	79	
	>4 cm	13	3	
Caspsule invasion	Absent	59	59	0.120
	Present	11	16	
	Extracapsular	53	34	
Intrathyroidal dissemination	Absent	105	95	0.693
	Present	18	14	
T staging	pT1	55	60	0.252
	pT2	11	12	
	pT3	38	27	
	pT4	19	10	
N staging	pN0	39	48	**0.002** *
	pN1a	35	41	
	pN1b	49	20	
M staging	M0	123	107	0.220
	M1	0	2	
Total thyroidectomy	Not done	95	81	0.603
	Done	28	28	
Lymph node dissection	Not done	3	12	**0.003** *
	CCND^a^ only	55	59	
	CCND with MRND^b^	65	38	

^a^ CCND, central compartment node dissection.

^b^ MRND, modified radical neck dissection.

* Significantly different by the χ^2^ test.

### 2.3 Cell culture, transfection and drugs treatment and infection

We cultured a normal thyroid cell line (Nthy‐ori 3‐1, ECACC), ATC cell line (8505C, DSMZ) and PTC cell line (TPC‐1, Fenghui Biotechnology) cells with RPMI‐1640 + 10% FBS medium. Whereas, 293T cells (ATCC) were cultured in DMEM + 10% FBS medium.

According to manufacturer instructions, cells were transfected with siRNAs (RiboBio Biotechnology, Guangzhou, China), HA‐BUB1 plasmids or FLAG‐KIF14 plasmids (Weizhen Biotechnology, Shandong, China) with jetPRIME (Polyplus, Berkeley, CA, USA). The siRNAs sequences were: GCAACAACAATACAGGTTATT for BUB1 siRNA#1, ACCAGTGAGTTCCTATCCAAA for BUB1 siRNA#2, CGGCAAGAAATAACATCCTTA for KIF14 siRNA#1 and GCGATTAGAAATGGAGACATT for KIF14 siRNA#2. We also treated cancer cells with 0 uM‐50 uM BUB1 inhibitor BAY‐1816032 to determine the IC_50_ value. Both cells were then incubated with IC_50_ value in subsequent in vitro experiments.

BUB1 knockdown and overexpression lentiviruses were purchased from ABM (Richmond, Canada) and the shRNA sequence was GCAACAACAATACAGGTTATT. Puromycin was utilized to select stable 8505C cells after infecting the lentivirus for 48 h.

### 2.4 Cell viability assay

The cells (2 × 10^3^ cells/well) transfected with siRNA or incubated with BAY‐1816032 were maintained for 48 h in 96‐well plates. Subsequently, Cell Counting Kit‐8 (CCK‐8; Fdbio, Hangzhou, China) was utilized to measure OD450 values.

### 2.5 Colony‐formation assay

Treated cells (1 × 10^3^ cells/well) were seeded into six‐well plates with a 3‐day change of the fresh medium. After maintenance for up to 10 days, colonies were fixed and dyed via a crystal violet solution. Cell colony numbers were counted using ImageJ software (https://imagej.nih.gov/ij/).

### 2.6 Flow cytometry

Cell cycle distribution was performed using flow cytometry. Samples were stained (CCS012, MultiSciences, Hangzhou, China) in terms of the manufacturer's instructions. The analysis flow chart of DNA content was recorded on the CytoFLEX cytometer (Beckman‐Coulter, USA) machine.

### 2.7 Transwell assay, wound healing assay

Transwell assays and wound healing assays were performed as previously described.^34^ For transwell assays, cells treated with siRNA or BAY‐1816032 were plated in the upper transwell chamber in the presence (invasion) or absence (migration) of Matrigel® (Cat. 356234, BD Bioscience, USA). Then cells were fixed with 4% paraformaldehyde and stained with 0.1% crystal violet after 48 h. We then dissolved the product in 33% glacial acetic acid and measured the absorbance at 560 nm using an American BioTek Synergy LX multi‐mode reader. Rate of invasion or migration: average OD of treated cells/average OD control units ×100%.^35^

### 2.8 qRT‐PCR, Western blots (WB) and co‐immunoprecipitation (co‐IP) assays

We performed qRT‐PCR, WB and co‐IP as previously described.^36^ The primers were as follows: BUB1, upstream primer: 5′‐TGGGACTGTTGATGCTCCAAACT‐3′, downstream primer: 5′‐GGAACTCACTGGTTTAGAAAGCCCAG‐3′; KIF14, upstream primer: 5′‐CGGAACAAGCAAACCAAAGGAGTG‐3′, downstream primer: 5′‐GCAGCGGGACTAATCGTAGCAATC‐3′; GAPDH, upstream primer: 5′‐GCACCGTCAAGGCTGAGAAC‐3′, downstream primer: 5′‐TGGTGAAGACGCCAGTGGA‐3′.

For WB, the primary antibodies included: BUB1 (A18053, ABclonal, Wuhan, China), KIF14 (26000‐1‐AP, Proteintech), p‐H3 (53,348, CST, USA), Flag (AE005, ABclonal), HA (AE105, ABclonal), GAPDH (10494‐1‐AP, Proteintech).

For co‐IP, the cell lysates were immunoprecipitated using anti‐BUB1 antibodies (sc‐365,685, Santa Cruz) or anti‐HA‐tag antibodies (AE105, ABclonal).

### 2.9 Immunohistochemistry (IHC) and immunofluorescent (IF) staining

We performed IHC and IF staining as previously reported.^27^ The primary antibody for IHC was the BUB1 antibody (YT0547, ImmunoWay, China). Immunoreactivity scores (IRS) of BUB1 were calculated as the product between the score for the staining positivity and intensity.^37^ 0–6 final scores indicated low BUB1 expression and 6–12 final scores indicated high BUB1 expression. The primary antibody for IF was the α‐tubulin antibody (C1051S, Biyuntian, Hangzhou, China). Treated cells were seeded (3 × 10^4^ cells/well) into four‐well confocal plates (D35C4‐20‐1‐N, FEIYUBIO, Nantong, China), and a Leica TCS SP8 confocal microscope was performed for confocal imaging.

### 2.10 Xenograft tumour model and zebrafish tumour metastasis model

The animal experiment protocol was the same as before.^36^ An assessment of body weight and tumour volume was carried out every 6 days. We used a similar method to assess the activity of BAY‐1816032. Two weeks later, mice were randomly allocated to two groups with similar tumour sizes: the control and BAY‐1816032 group. Normal saline or BAY‐181032 was orally administered every day for 24 days at a dose of 100 mg/kg.

### 2.11 Phos‐tag analysis

Phos‐tag analyses for the detection of phosphorylation of KIF14 were completed according to manufacturer instructions (F4002, APExBIO). The antibody used was an anti‐KIF14 antibody (26000‐1‐AP, Proteintech).

### 2.12 Cell synchronization

Nocodazole (100 ng/mL) was administered to cells for 16 h (−16 h). We then added the siRNAs to the media 8 h before synchronization was completed (−8 h). After 8 h of new nocodazole‐free media, the cells were released from synchronization (0 h).

### 2.13 Genomic sequencing

The sample preparation for whole exome sequencing (WES) required 0.6 μg of genomic DNA. The sequencing library was formed by Agilent SureSelect Human All‐Exon V6 (Agilent, USA). Lastly, we sequenced 150‐bp paired‐end by an Illumina Novaseq6000 system through the above‐generated DNA libraries.

### 2.14 Giemsa staining

Cells were stained using a Giemsa staining kit following the manufacturer's instructions (BBI Life Sciences, Shanghai, China).

### 2.15 In vitro kinase assay

For the in vitro kinase assay, 200 ng of recombinant KIF14 (WT) or mutant (KIF14^ΔSer1292^) protein and 100 ng of purified BUB1 were incubated for 1 h at 30°C in a kinase assay buffer containing 500 mM ATP. SDS sample buffer was used to terminate the reaction. Every sample was boiled and analysed using Phos‐tag analysis.

### 2.16 Statistical analysis

Statistics analyses were employed by referring to the previous protocols in GraphPad Prism v8.0 and SPSS v25.0 (SPSS).^36^

## 3 RESULTS

### 3.1 BUB1 was significantly elevated in aggressive TC, and its expression level was related to adverse outcomes

**FIGURE 1:**
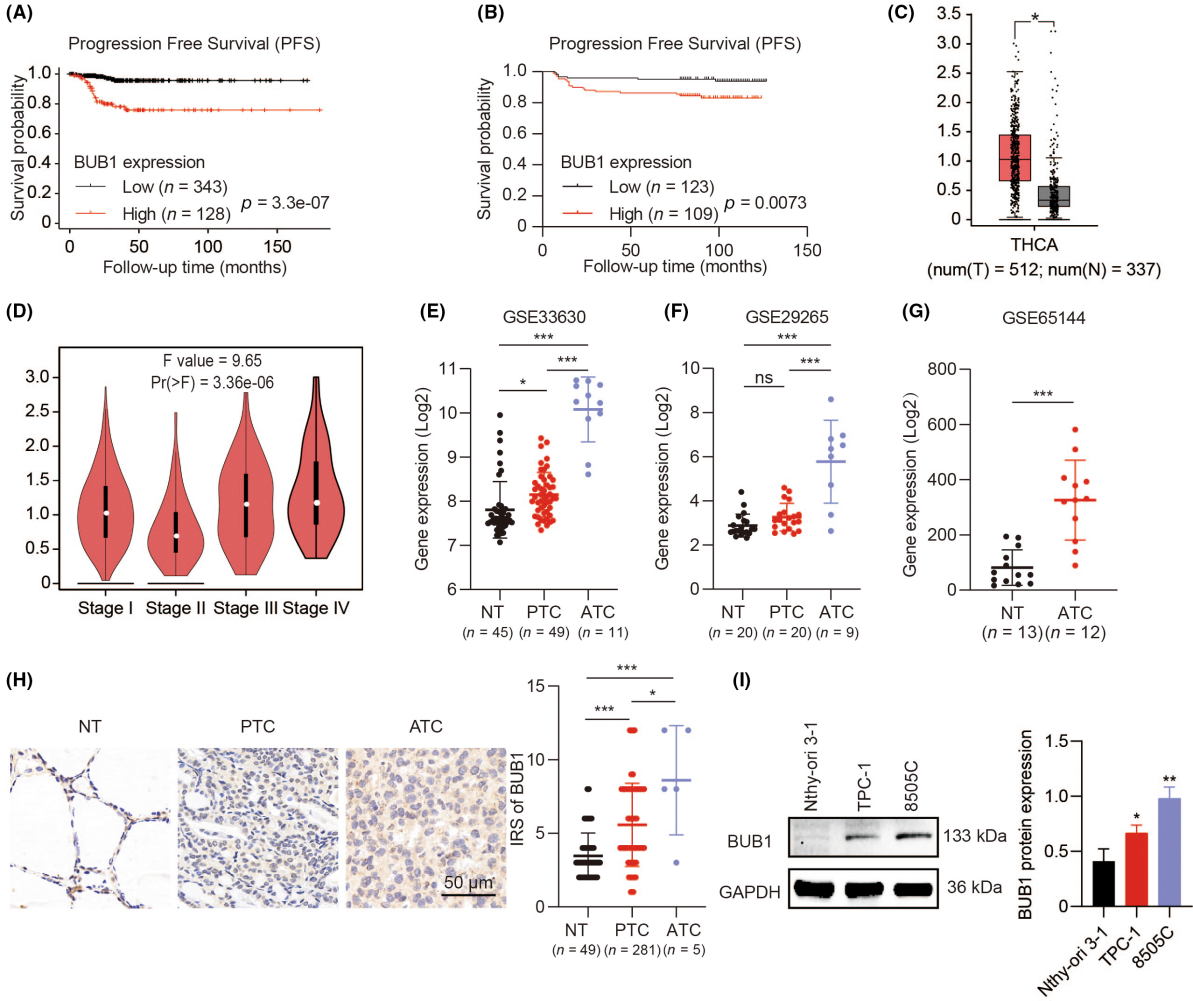
BUB1 expression with its clinical significance in TC. (A) Association between PFS and BUB1 expression (RNA‐seq) in TC from Kaplan–Meier plotter database. (B) Correlation between BUB1 expression (IHC) and PFS based on a cohort of a total of 232 samples of PTC from tissue microarrays. (C, D) BUB1 expression in normal and TC samples and the relevance with tumour stage in GEPIA database. (E–G) Expression of BUB1 in TC from datasets GSE33630, GSE29265 and GSE65144. (H) BUB1 expression was examined in normal, PTC and ATC tissues through immunohistochemistry staining. (I) BUB1 protein levels among TC cells were detected using WB assays. (*p < 0.05, **p < 0.01, ***p < 0.001; mean ± standard deviation (SD)).

To examine the possible relationship between BUB1 and ATC progression, a survival analysis was performed for PFS. Patients with TC overexpressing BUB1 correlated with worse outcomes (*p* < 0.001) (Figure 1A). In addition, high BUB1 expression was an effective PFS predictor (Table 2). Based on an independent cohort of 232 TC samples, IHC staining of BUB1 was performed, which further identified high BUB1 expression was associated with poor outcomes (*p* = 0.0073) (Figure 1B).

**TABLE 2:** Univariate and multivariate Cox regression analysis of BUB1 expression with patient prognosis.

Variable	Univariate analysis		Multivariate analysis	
	HR (95% CI)	*p*‐Value	HR (95% CI)	*p*‐Value
Age (years)	0.974 (0.943–1.007)	0.118		
Gender	0.843 (0.352–2.018)	0.701		
Histological variants	0.308 (0.042–2.280)	0.249		
Bilaterality	2.766 (1.242–6.160)	**0.013** *	2.314 (0.660–8.113)	0.190
Tumour number	1.316 (0.582–2.979)	0.510		
Maximal tumour diameter	1.313 (1.056–1.633)	**0.014** *	1.168 (0.786–1.734)	0.443
Capsule invasion	1.756 (1.131–2.725)	**0.012** *	1.535 (0.870–2.709)	0.139
Intrathyroidal dissemination	1.641 (0.616–4.373)	0.322		
TNM staging	1.174 (0.531–2.595)	0.692		
Total thyroidectomy	2.709 (1.228–5.976)	**0.014** *	0.085 (0.017–0.433)	**0.003** *
Lymph node dissection	1.941 (0.932–4.044)	0.077		
Iodine radiotherapy	16.975 (7.286–39.550)	**0.000** *	35.940 (11.539–111.942)	**0.000** *
BUB1 expression	3.101 (1.294–7.431)	**0.011** *	3.117 (1.161–8.367)	**0.024** *

Abbreviation: HR, Hazard ratio.

* Statistical significance.

Data from the GEPIA database showed that a prominent upregulation of BUB1 was observed in TC (Figure 1C), particularly in aggressive TC (Figure 1D). According to three GEO datasets, BUB1 displayed greater expression in ATC in contrast with PTC and normal counterparts (Figure 1E–G). Consistently, BUB1 exhibited a high expression in ATC tissues and ATC cell lines, as revealed by IHC and WB, respectively (Figure 1H,I).

### 3.2 BUB1 expression level correlated with ATC cell invasive phenotype

**FIGURE 2:**
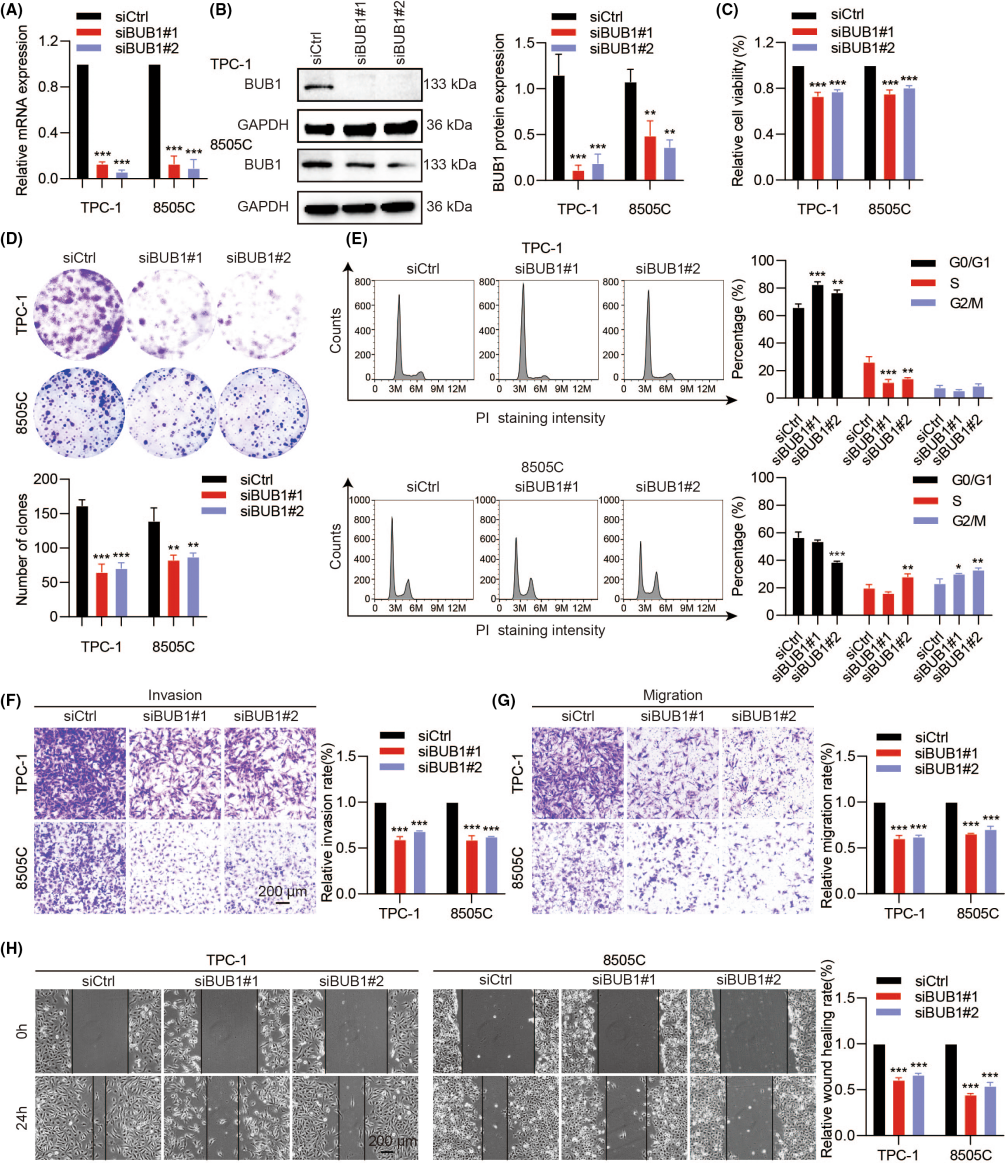
Reduced expression of BUB1 in TC cells suppresses invasive phenotype. (A, B) Verification of BUB1‐siRNA silencing efficacy at mRNA and protein levels. Cell viability assays (C) and colony‐formation assays (D) were measured after BUB1 silencing. (E) The cell cycle of BUB1 silencing in TC cells was determined using flow cytometry. (F–H) Invasion, migration and wound healing assays of BUB1 silence were conducted in TC cells. (*p < 0.05, **p < 0.01, ***p < 0.001; mean ± SD).

To examine whether BUB1 overexpression contributed to ATC's invasive phenotype, we first knocked down it with high efficiency (Figure 2A,B). Proliferation was decreased in siBUB1 transfected ATC cells, as demonstrated by a cell viability assay and a clonogenic assay (Figure 2C,D). Moreover, cell cycle arrests in the G0/G1 phase of TPC‐1 and the G2/M phase of 8505C were discovered via flow cytometry (Figure 2E). However, overexpression of BUB1 decreased the proportion of the G0/G1 phase in TPC‐1 cells, and this phenotype was partially reversed when BUB1 was simultaneously silenced (Figure S1A). Furthermore, the invasion and migration abilities were significantly attenuated in TPC‐1 and 8505C cells after silencing of BUB1 (Figure 2F,G). Similarly, BUB1 knockdown markedly suppressed wound healing in both cell lines (Figure 2H).

### 3.3 Tumorigenesis and metastasis in ATC cells were markedly inhibited by the knockdown of BUB1

**FIGURE 3:**
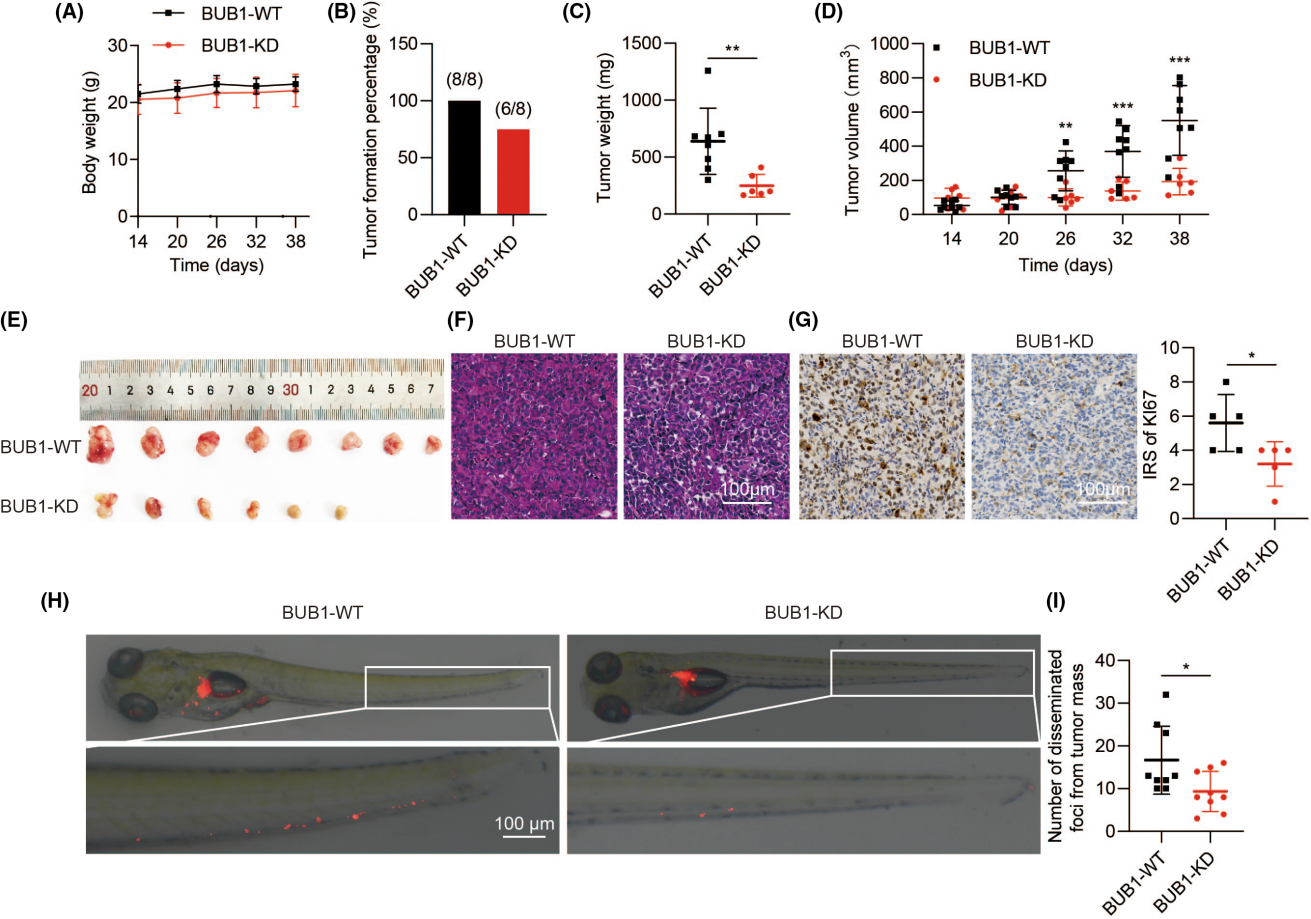
Nude mice with ATC xenografts exhibit decreased tumorigenesis and slower tumour growth after BUB1 knockdown. The xenografts were developed from BUB1‐WT or BUB1‐KD 8505C cells. (A–E) Body weight, tumour formation rate, tumour weight and tumour volume. (F) H&E staining of tumour tissues. (G) Detection of Ki67 expression performed by immunohistochemistry. (H) The zebrafish xenograft model was employed to evaluate the metastatic ability of 8505C cells, after BUB1 knockdown. (I) Quantification of disseminated tumour foci. (*p < 0.05, **p < 0.01, ***p < 0.001; mean ± SD).

We then assessed if BUB1 expression affected tumour growth in vivo. Although the body weight of each group was similar, the tumour formation was significantly reduced in animals implanted with BUB1 knockdown cell‐derived xenografts (6/8) (Figure 3A,B). Excitingly, the mean tumour weight was obviously lower in the BUB1‐KD group (248.6 ± 99.8 mg) than that in the BUB1‐WT group (638.3 ± 290.1 mg) (*p* < 0.01) (Figure 3C). Consistently, the average tumour volume in the BUB1‐KD group (192.7 ± 78.0 mm^3^) was also nearly 65% smaller than that in the BUB1‐WT group (550.8 ± 204.3 mm^3^) (Figure 3D,E). H&E staining indicated that the BUB1‐KD group of tumour tissues contained fewer tumour cells than tumours in the BUB‐WT group mice (Figure 3F). Compared with the BUB1‐WT group, the proliferation marker Ki67 in the BUB1‐KD group was significantly decreased (Figure 3G).

Next, we established a zebrafish model to evaluate the effects of BUB1 on ATC metastasis. BUB1 knockdown significantly inhibited the ability of metastasis on 5 dpf in the ATC cell‐derived xenograft (Figure 3H). Compared with BUB1‐WT cells, the BUB1‐KD cells led to fewer disseminated tumour foci (Figure 3I).

### 3.4 Effects of BUB1 inhibitor on aggressive phenotypes of ATC cells

**FIGURE 4:**
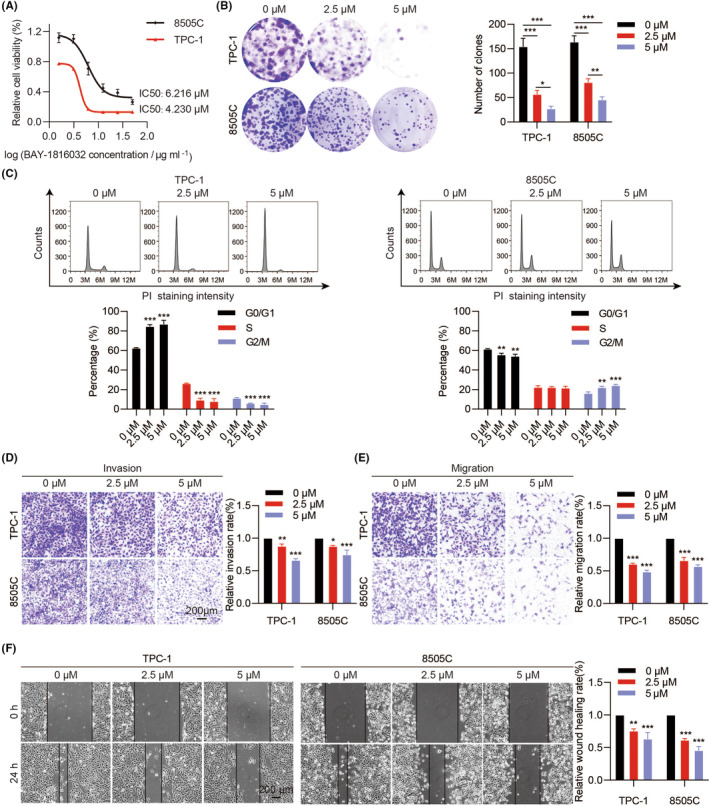
The effect of tumour inhibition by BUB1 inhibitor BAY‐1816032 in vitro. (A) We exposed cancer cells to varying BAY‐1816032 concentrations (0, 1.5625, 3.125, 6.25, 12.5, 25 and 50 μM) for 48 h. CCK‐8 assays were employed to investigate cell viability. Colony‐formation assays (B), cell cycle (C), invasion (D), migration (E) and wound healing assays (F) were detected after being treated with BAY‐1816032 in a dose‐dependent manner (0, 2.5, 5 μM). (*p < 0.05, **p < 0.01, ***p < 0.001; mean ± SD).

The ATC cells viability was decreased by BAY‐1816032 treatment, and the IC_50_ values were 4.230 μM in TPC‐1 cells and 6.216 μM in 8505C cells, respectively (Figure 4A). Therefore, we chose IC_50_ values as the optimal concentrations for the subsequent experiments. Our results revealed that BAY‐1816032 can significantly decrease the colony‐forming capacities (Figure 4B). In addition, BAY‐1816032 caused cell cycle arrests similar to siRNAs (Figure 4C). Furthermore, treatment with BAY‐1816032 dramatically impeded ATC‐cell invasion and migration in a dose‐dependent manner (Figure 4D,E). An obvious, dose‐dependent reduction in the scratch healing area of the TPC‐1 and 8505C cells was also observed (Figure 4F).

### 3.5 Anti‐tumour activity of BUB1 inhibitor in ATC

**FIGURE 5:**
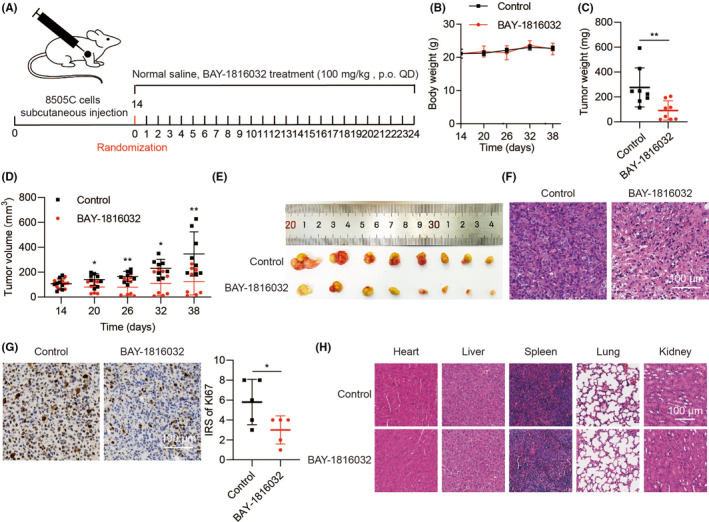
The activity of BUB1 inhibition by BAY‐1816032 for tumour growth in ATC in vivo. (A) A BAY‐1816032 oral administration anti‐tumour experiment performed with a mouse xenograft model is shown. We divided the xenografts into the control group and the BAY‐1816032 group after 2 weeks. (B–E) Body weight, tumour weight and tumour volume. (F–G) H&E staining and immunohistochemistry of Ki67 in each group of mice tumour tissues. (H) H&E staining in each group of mice hearts, livers, spleens, lungs and kidneys. (*p < 0.05, **p < 0.01; mean ± SD).

Next, we identified the anti‐ATC activity and safety of BAY‐1816032 in vivo. Mice were administered with normal saline or BAY‐1816032 orally every day for 24 days at a dose of 100 mg/kg (Figure 5A). Body weights of each group were not significantly different, demonstrating the safety of the drug (Figure 5B). We noted that the tumour weights in the administration group (91.4 ± 77.4 mg) were obviously lower compared with the saline group (276.3 ± 157.5 mg) (Figure 5C). And the tumour volumes of mice treated with BAY‐1816032 (125.4 ± 108.3 mm^3^) were also markedly smaller than that in the saline group (346.3 ± 176.9 mm^3^), a decrease of near 2.76 folds (Figure 5D,E). Besides, the tumour tissue of the experimental group contained fewer tumour cells and lower Ki67 expression than (Figure 5F,G). Histological analyses of tissues from the heart, liver, spleen, lungs and kidneys did not show obvious pathological alterations upon BAY‐1816032 administration (Figure 5H).

### 3.6 High expression of BUB1 evokes CIN in ATC

**FIGURE 6:**
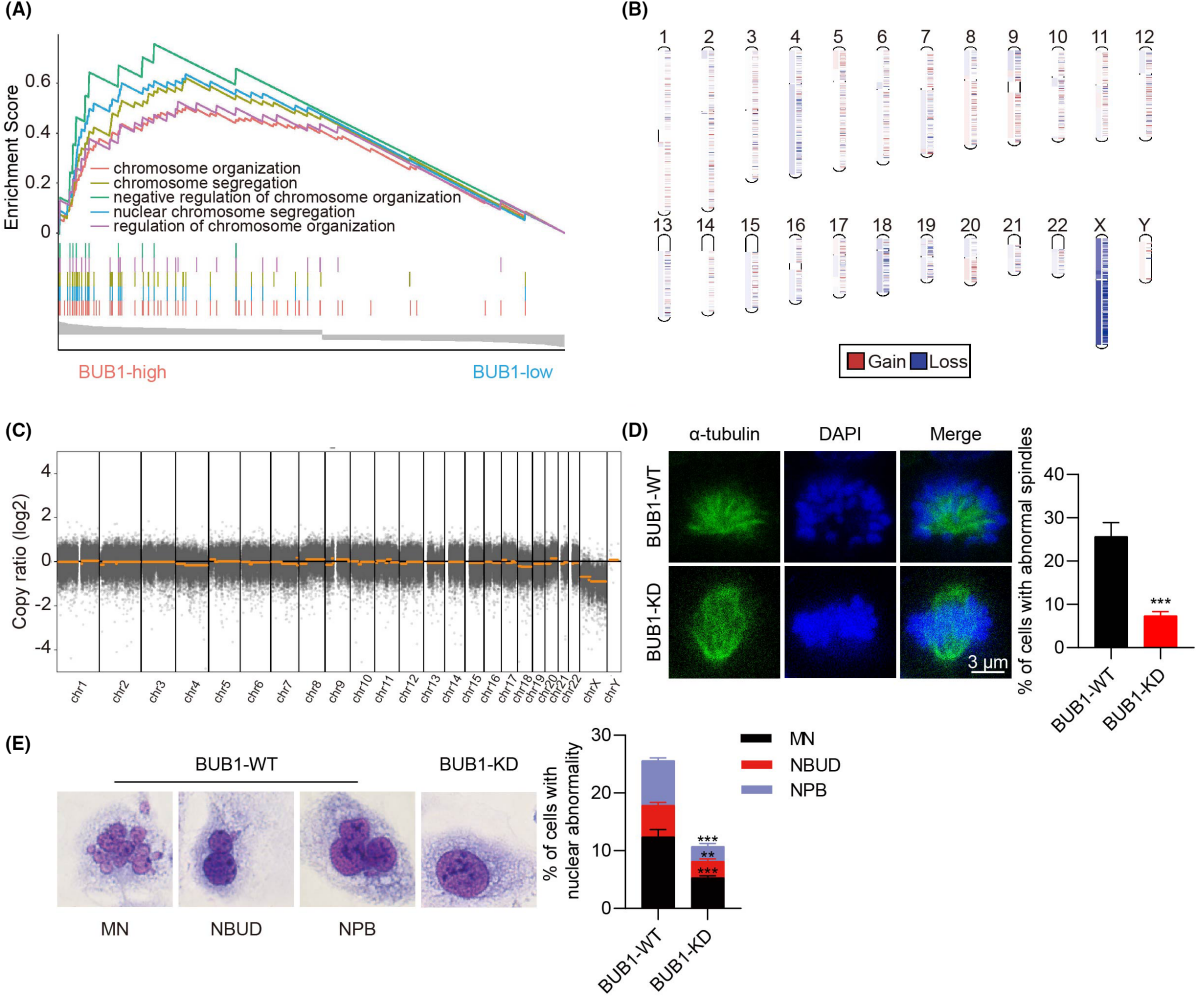
BUB1 evokes CIN in ATC. (A) A biological process enrichment GO annotation was performed using GSEA grouped by expression of BUB1. (B, C) Gain or loss of the chromosomal segments in 8505C cells after BUB1 knockdown was detected by WES. (D) Immunofluorescent staining of α‐tubulin to detect the formation of abnormal spindle after BUB1 knockdown. (E) The nuclear abnormalities in BUB1‐KD 8505C cells, as revealed by the Giemsa staining, include nucleoplasmic bridges (NPB), micronucleus (MN) and nuclear buds (NBUD). (**p < 0.01, ***p < 0.001; mean ± SD).

Given that BUB1 is essential for ensuring proper chromosome separation to maintain genomic stability during cell division, we explored whether BUB1 contributes to ATC CIN. GSEA revealed that increased expression of BUB1 correlated significantly with the organization and segregation of chromosome signalling (Figure 6A). We subsequently performed WES to directly evaluate the impact of BUB1 knockdown in ATC on chromosomal composition from a genomic perspective. It was shown that BUB1‐KD 8505C cells had obvious increases and decreases of multiple chromosome segments in comparison with BUB1‐WT cells. Among them, chromosome 4, 18 and X losses were the most obvious (Figure 6B,C). Since cancer cells are easier to exhibit chromosome segregation errors due to abnormal mitotic spindles, IF staining of α‐tubulin was further employed to assess the degree of CIN evoked by BUB1.^38^, ^39^ Our results revealed that BUB1‐KD decreased the percentage of abnormal mitotic spindles in 8505C cells (Figure 6D). Among them, most BUB1‐KD cells displayed normal chromosome alignment and spindle morphology. However, in BUB1‐WT cells, spindle formation was disrupted and asymmetric spindle including monopolar spindles was observed, thus leading to aberrant chromosome segregation. Similar findings have been reported in previous studies conducted by other researchers.^40^, ^41^, ^42^, ^43^ Next, the impact of BUB1 knockdown on abnormal nuclear morphology was investigated by Giemsa staining. The presence of nucleoplasmic bridges (NPB), nuclear buds (NBUD) and micronucleus (MN), indicators of CIN,^44^ reduced in the 8505C cells with BUB1 knockdown (Figure 6E).

### 3.7 BUB1 promotes CIN through interacting with KIF14

**FIGURE 7:**
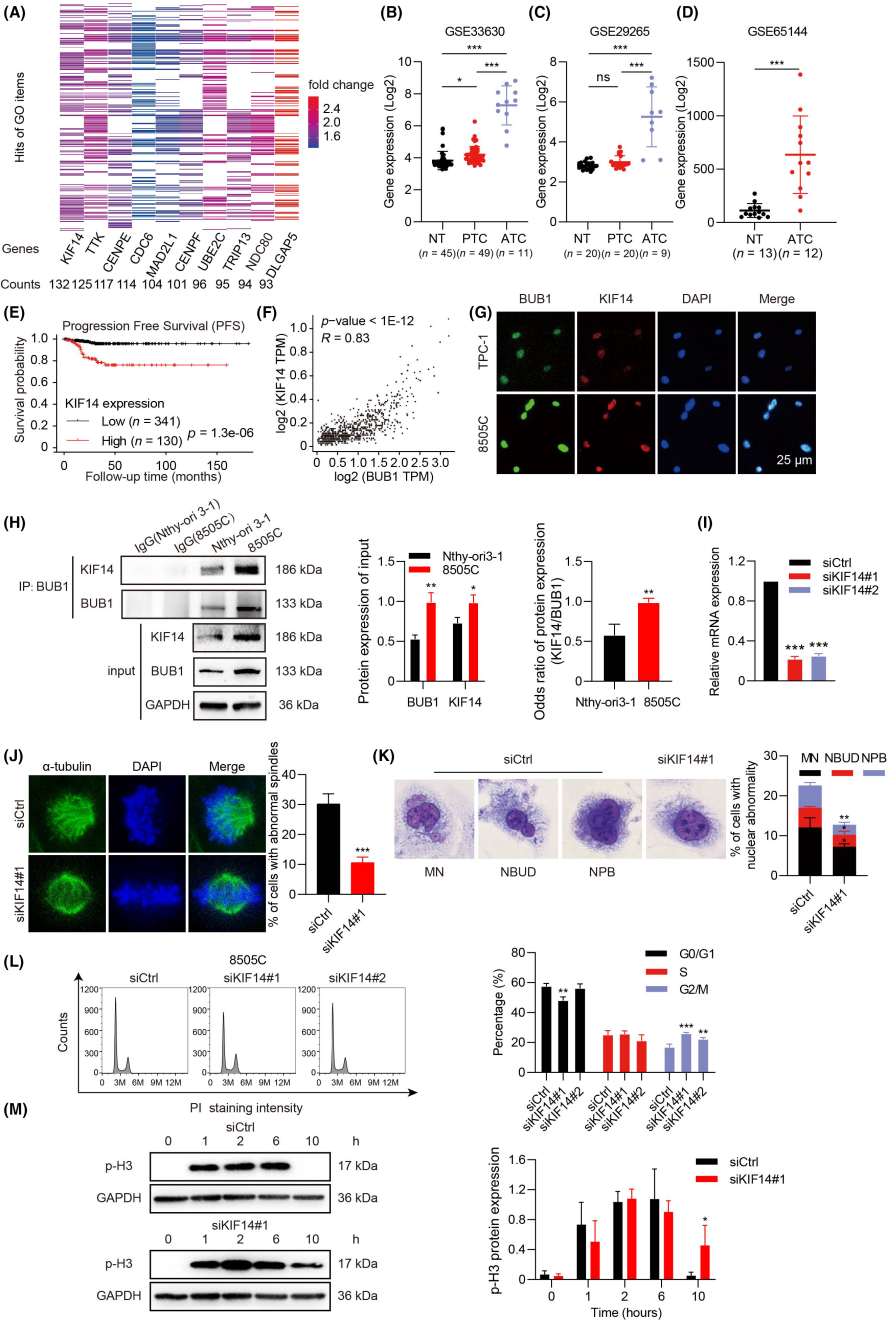
BUB1 promotes CIN through interacting with KIF14 in ATC. (A) All significantly enriched gene ontology‐biological process terms from the top 10 enriched genes between high and low BUB1 expression were displayed on a heatmap. KIF14 expression (B–D) and its clinical significance (E) in TC. (F) Correlation analysis of BUB1 and KIF14 expression levels in the GEPIA database. (G) Immunofluorescence assays were conducted to verify the colocalization of BUB1 and KIF14. (H) Lysates derived from Nthy‐ori 3‐1 and 8505C cells were utilized for co‐IP of BUB1 and KIF14. The odds ratio of KIF14/BUB1 protein expression was calculated as$\;\frac{\mathrm{KIF}14\left(\mathrm{IP}\right)}{\mathrm{KIF}14\left(\text{input}\right)}÷\frac{\mathrm{BUB}1\left(\mathrm{IP}\right)}{\mathrm{BUB}1\left(\text{input}\right)}$. (I) Verification of KIF14 siRNA silencing efficacy at the mRNA level in 8505C cells. Immunofluorescent staining (J), Giemsa staining (K), and cell cycle distribution analysis (L) were performed after silencing of KIF14. (M) Temporal variation in p‐H3 examined by WB. (*p < 0.05, **p < 0.01, ***p < 0.001; mean ± SD).

To identify the mechanism by which BUB1 influenced CIN, GSEA data was excavated to probe the BUB1 downstream targets. High expression of BUB1 was closely associated with 252 biological processes, and KIF14 was the most frequently enriched gene in the above pathways (Figure 7A). Furthermore, KIF14 was elevated in ATC, and its expression level was related to poor outcome (Figure 7B–E). Additionally, the expression of BUB1 and KIF14 in ATC showed a significant positive correlation (Figure 7F).

The significant colocalization of BUB1 and KIF14 in the nuclear regions was further observed by IF staining (Figure 7G). The co‐IP experiment also showed that BUB1/KIF14 complex was increased in 8505C cells than that in Nthy‐ori 3‐1 cells (Figure 7H). KIF14 was then knocked down in 8505C cells by siRNA (Figure 7I), resulting in the reduction of CIN (Figure 7J,K). Similarly, KIF14 silencing induced G2/M phase arrests in 8505C cells (Figure 7L). In addition, we used nocodazole to synchronize 8505C cells during the G2/M phase and measured mitosis length. Significantly, the silence of KIF14 delayed mitotic exit in cells, as indicated by the decrease in histone H3 phosphorylation levels (10 h versus 6 h), an indicator of M‐phase,^45^ revealing that KIF14 knockdown induced a prolonged mitotic arrest. (Figure 7M).

### 3.8 BUB1 induces CIN of ATC by phosphorylating KIF14 at Ser^1292^

**FIGURE 8:**
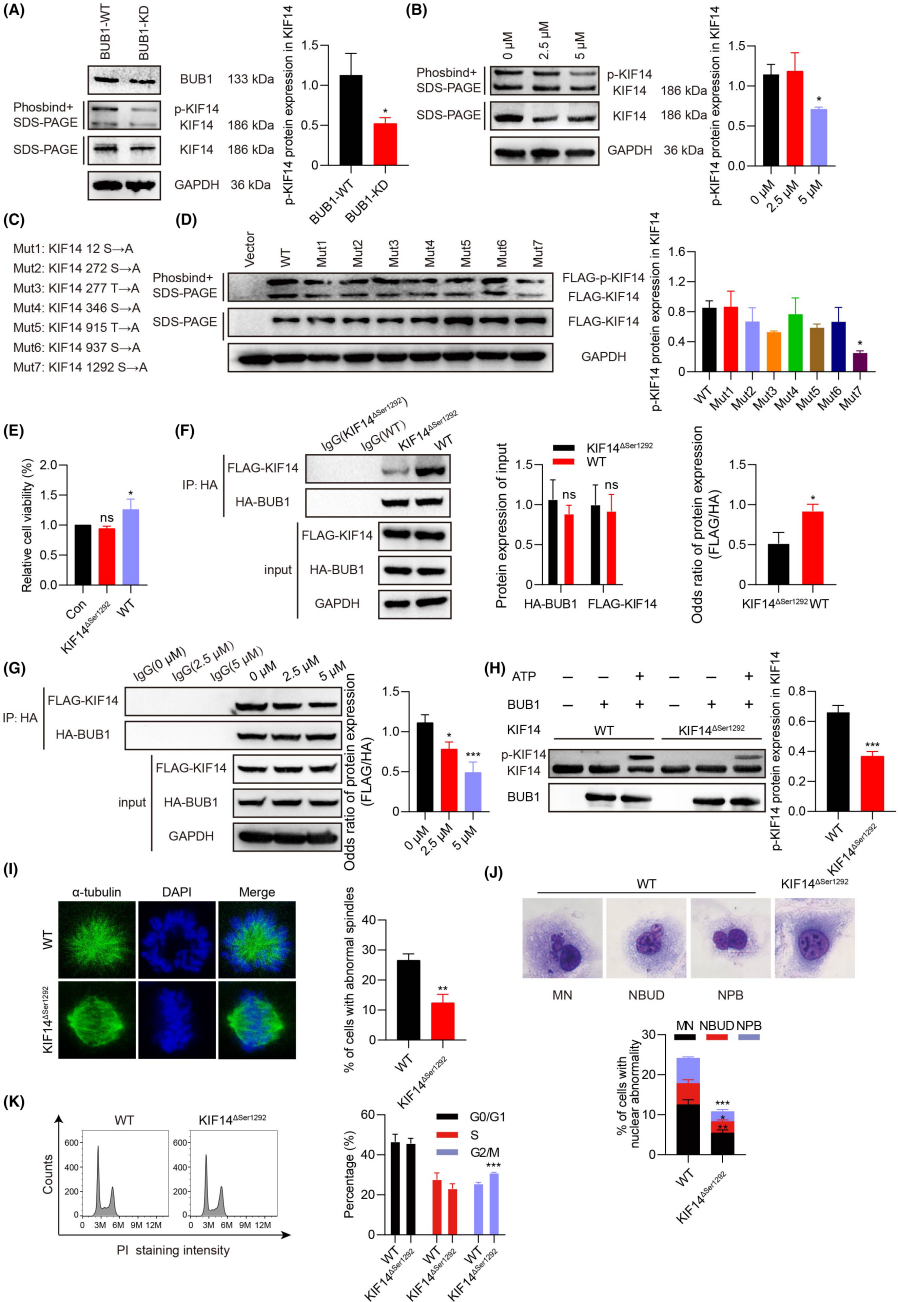
BUB1 induces CIN of ATC through phosphorylation of KIF14 at Ser^1292^. (A, B) The effects of BUB1 knockdown and BUB1 inhibitor BAY‐1816032 on KIF14 phosphorylation were detected using Phos‐tag assays. SDS‐PAGE gels were used to conduct WB for total KIF14. The expression level of p‐KIF14 protein was determined by its ratio to the total expression level of KIF14 protein. (C) The potential phosphorylation sites of KIF14 were retrieved from the UniProt website and mutated to alanine (Ala), respectively. (D) The effects of Flag‐tagged KIF14 mutant plasmids on its phosphorylation were conducted by a Phos‐tag assay in 8505C cells. (E) Cell viability assays were measured after transfecting with Flag‐tagged KIF14 mutant plasmids. (F) Lysates derived from 293T cells were utilized for co‐IP of HA‐BUB1 and FLAG‐KIF14. The cells were all transfected with HA‐BUB1 but were transfected with KIF14^ΔSer1292^ mutant or WT plasmids, respectively. The odds ratio of FLAG‐KIF14/HA‐BUB1 protein expression was calculated as$\;\frac{\text{FLAG}-\mathrm{KIF}14\left(\mathrm{IP}\right)}{\text{FLAG}-\mathrm{KIF}14\left(\text{input}\right)}÷\frac{\mathrm{HA}-\mathrm{BUB}1\left(\mathrm{IP}\right)}{\mathrm{HA}-\mathrm{BUB}1\left(\text{input}\right)}$. (G) Lysates derived from 293T cells were utilized for co‐IP of HA‐BUB1 and FLAG‐KIF14. The cells were first transfected with HA‐BUB1 and FLAG‐KIF14 WT plasmids and then treated with BAY‐1816032 in a dose‐dependent manner. The odds ratio of FLAG‐KIF14/HA‐BUB1 protein expression was calculated as$\;\frac{\text{FLAG}-\mathrm{KIF}14\left(\mathrm{IP}\right)}{\text{FLAG}-\mathrm{KIF}14\left(\text{input}\right)}÷\frac{\mathrm{HA}-\mathrm{BUB}1\left(\mathrm{IP}\right)}{\mathrm{HA}-\mathrm{BUB}1\left(\text{input}\right)}$. (H) The in vitro kinase assays. BUB1 kinase was mixed with purified recombinant KIF14 or KIF14^ΔSer1292^ protein, and KIF14 phosphorylation was examined by Phos‐tag assays. The expression level of p‐KIF14 protein was determined by its ratio to the total expression level of KIF14 protein. Immunofluorescent staining (I), Giemsa staining (J) and cell cycle distribution analysis (K) were performed after overexpression of KIF14^ΔSer1292^ mutant (*p < 0.05, **p < 0.01, ***p < 0.001; mean ± SD).

Since BUB1 is a protein kinase that regulates the post‐translational modification of proteins, we investigated whether KIF14 is a substrate of BUB1. The results of the Phos‐tag assays determined that both knockdown of BUB1 and inhibition of BUB1 activity caused the downregulation of phosphorylated KIF14 in 8505C cells (Figure 8A,B). To make it clear the specific BUB1 phosphorylation site in KIF14, we individually generated phosphorylation‐null mutants of KIF14 by site‐directed mutagenesis of Ala (Figure 8C). The results showed that Ser^1292^ site mutation led to evident downregulation of phosphorylation level of KIF14 (Figure 8D). Proliferation was increased in KIF14 WT plasmid transfected 8505C cells, as demonstrated by a cell viability assay. Moreover, no differences were discovered between the control group and the KIF14^ΔSer1292^ mutant group. Therefore, only the WT group and the KIF14^ΔSer1292^ group were selected for use in subsequent experiments (Figure 8E). The co‐IP experiment further indicated that BUB1/KIF14 complex was decreased in 293T cells after the transfection of the KIF14^ΔSer1292^ mutant (Figure 8F). To further investigate the role of BUB1 as a kinase in the phosphorylation of KIF14, we transfected HA‐BUB1 and FLAG‐KIF14 plasmids into 293T cells and treated with BUB1 inhibitor BAY‐1816032. The results displayed that BAY‐1816032 significantly attenuated the formation of the BUB1/KIF14 complex (Figure 8G). An in vitro kinase assay was further performed to determine whether BUB1 directly phosphorylated KIF14. Purified recombinant BUB1 protein was incubated with purified recombinant KIF14 or its mutant form, KIF14^ΔSer1292^. The phosphorylation band of KIF14 appeared after incubation with BUB1 kinase and purified recombinant KIF14 protein, and compared with WT, the purified recombinant KIF14^ΔSer1292^ protein downregulated the proportion of p‐KIF14 by nearly 1.79 folds (*p* < 0.001) (Figure 8H). Taken together, these indicate that Ser^1292^ was an important site for BUB1 phosphorylation modification of KIF14.

Interestingly, we discovered that the KIF14^ΔSer1292^ mutant affected CIN, with a reduced percentage of abnormal mitotic spindles and abnormal nuclear morphology (Figure 8I,J). In addition, the G2/M phase arrests occurred after the transfection of the KIF14^ΔSer1292^ mutant (Figure 8K). Combined, these results suggested that BUB1 is involved in ATC CIN through the phosphorylation of KIF14 at Ser^1292^.

## 4 DISCUSSION

CIN is considered a hallmark of cancer that contributes to tumorigenesis and the progression of cancer.^12^, ^46^ Previous research has revealed that there is an overexpression of a gene cluster associated with cell proliferation and genetic instability in thyroid cells during anaplastic transformation.^18^ However, the molecular mechanism that contributes to the high CIN in ATC remains uncertain. Our research identified the SAC family member BUB1 as a key regulator of CIN in ATC. It was found that BUB1 expression levels were higher in ATC compared to PTC, which correlated with poor PFS. Further, both knockdown and inhibition of BUB1 significantly induced arrests of the cell cycle and attenuated growth and metastasis in ATC cells and xenografts. These BUB1‐mediated effects were associated with the maintenance of CIN through interacting with KIF14. Phosphorylation of KIF14 at Ser^1292^ by BUB1 was required for aberrant mitosis of ATC cells.

BUB1 is a critical protein of SAC that is strongly expressed in several malignancies and correlates with poor prognosis.^25^, ^47^, ^48^ BUB1 regulates STAT3 signalling through transcriptional activation to facilitate bladder cancer progression and proliferation.^49^ In liver cancer, BUB1 promotes cell proliferation by activating the phosphorylation of SMAD2.^48^ According to our results, BUB1 levels in ATC were also obviously upregulated and can be regarded as a poor prognostic clinical indicator. Furthermore, ATC expression levels were significantly higher compared to the less malignant PTC, indicating that BUB1 may contribute to ATC aggressiveness. We also verified BAY‐1816032, a new inhibitor of BUB1 catalytic activity that is biologically available, which promotes centromere cohesion and correction of attachment errors.^50^ It has been demonstrated in a clinical proof‐of‐concept study that BAY‐1816032 enhanced the efficacy of PARP inhibitors or taxanes and possibly overcame the resistance of cancer cells, including HeLa, prostate cancer and triple‐negative breast cancer, et al.^50^ Our nude mouse model confirmed this, suggesting an essential role of BUB1 in ATC. Considering that ATC is typically refractory to standard therapy, BUB1 possesses an attractive therapeutic potential for targeted therapy. Interestingly, our studies showed that knockdown and inhibition of BUB1 caused arrests of the G0/G1 phase in PTC cell line TPC‐1 and G2/M phase in ATC cell line 8505C. Previous reports have described a similar phenomenon, which was due to differences in cell cycle‐related protein expression between individual cancer cells. For instance, ailanthone upregulated p21 protein and downregulated cyclin E to induce G0/G1 phase arrests in B16 cells. It also upregulated p21 protein but downregulated cyclin B to induce G2/M phase arrests in A375 cells.^51^ Given that any mutations of regulatory mechanisms in the cell cycle can result in abnormal chromosome numbers or cell reproduction containing genetic mutations, the possible mechanism for BUB1 knockdown on the cell cycle would be worthy of investigation.^52^

The progression of cancer is caused by a series of dynamic molecular events resulting in aberrant phenotypes and genetic instability.^53^ ATC exhibits extensive gene expression alterations and cytogenetic abnormalities, which are significantly related to CIN and promote the progression of invasive disease.^16^, ^54^, ^55^, ^56^ Several mechanisms may contribute to the process of CIN, including ineffective/erroneous DNA repair, dysfunction/shortening of telomeres, defects in spindle assembly or chromosome segregation, etc.^57^ Among them, BUB1 expression abnormally affects spindle checkpoint function, thus causing CIN during mitosis.^58^ During cell mitosis, the SAC ensures proper and equal distribution of sister chromatids into daughter cells by inhibiting the onset of mitotic anaphase until all sister chromatids are appropriately aligned on the equatorial plate.^59^ Abnormal overexpression of BUB1 disrupts SAC function, leading to premature entry into mitotic anaphase when sister chromatids are not correctly arranged. Consequently, the unequal distribution of improperly aligned sister chromatids into daughter cells leads to aneuploid cells and CIN. Our research observed that abnormal overexpression of BUB1 impairs SAC function, forming asymmetric spindles, including monopolar spindles. This disrupts chromosome separation during mitosis and ultimately leads to CIN. Previous studies have reported similar spindle structures,^40^, ^41^, ^42^, ^43^ and demonstrated that elevated BUB1 expression in lymphoma cells causes aneuploidy through excessive activation of Aurora B kinase.^60^ These findings support our observations on BUB1's role in CIN and emphasize the importance of regulating its expression for mitotic progression. Our results indicated that BUB1 overexpression was the contributor to serving CIN in ATC cells versus PTC cells. Because PTC is considered an early step in the transition to ATC,^61^ whether BUB1 contributes to this progression should be investigated in future studies. Further, we noticed that chromosome X loss was most obvious in BUB1‐KD cells, and OPHN1, DOCK11 and CENPI were the most apparently changed genes in chromosome X (data not shown). OPHN1 overexpression was observed in prostate cancer (PCa) after androgen deprivation therapy, which promoted PCa progression and cell survival.^62^ Moreover, CENPI is related to adverse outcomes and promotes CIN in *ER*‐positive breast cancer.^63^ Given the cancer‐promoting effect of these genes, how BUB1 affects the changes of these chromosomes need further study.

The excavation of GSEA data indicated KIF14 participated in more than 50% (132/252) of high BUB1 connection biological processes, ranking first, indicating the close relationship with BUB1. The KIF14 protein belongs to the kinesin subfamily 3 locates on chromosome 1q32.1. It is an essential cytoskeletal protein responsible for cell cycle progression, mitotic spindle formation, cytokinesis completion and chromosomal alignment, segregation and congression.^40^, ^64^, ^65^, ^66^, ^67^, ^68^, ^69^, ^70^ It has been proved that KIF14 is frequently overexpressed in cancers, which correlates with adverse clinical prognoses, tumour metastases and recurrence.^71^, ^72^, ^73^, ^74^, ^75^, ^76^, ^77^ Importantly, KIF14 overexpression during tumorigenesis could result in rapid and error‐prone mitosis, thereby causing aneuploidy.^65^ In the present study, we confirmed for the first evidence that this is also true of ATC, and silence of KIF14 resulted in the reduction of CIN and induced G2/M phase arrests. KIF14 is critical for spindle microtubule formation and organization, ensuring proper chromosome segregation during cell division. As a downstream target of BUB1, the formation of asymmetric spindle, including the presence of monopolar spindles, resulting from abnormal KIF14 expression is therefore reasonable and expected.

The BUB1 kinase activity has been proved by two direct identified substrates. First, BUB1 phosphorylates CDC20 in vitro to play a direct role in SAC signalling.^78^ Importantly, BUB1 phosphorylates histone H2A at the conserved residue that corresponds to threonine121 in yeast, thereby establishing the binding site to MEI‐S32 and Shugoshin protein for recruiting chromosome passenger complex to centromere.^79^, ^80^, ^81^, ^82^ Nevertheless, how BUB1 acts as a kinase on the downstream signal in ATC has not been reported. KIF14 has been reported to function as a downstream target in malignancy. Its upregulation facilitates cell invasion and angiogenesis of glioma, whereas its downregulation decreases proliferation and increases apoptosis in retinoblastoma cells.^83^, ^84^ However, few studies have focused on its involvement in post‐translational modification. Notably, we identified KIF14 as a newly discovered substrate of BUB1. KIF14 was phosphorylated at Ser^1292^, and its phosphorylation‐null mutation disrupted the interaction with BUB1, thus eliminating the BUB1‐induced CIN signature. However, even if we identified that BUB1 indeed phosphorylated KIF14 Ser^1292^ in several experiments, and we confirmed the important role of KIF14 Ser^1292^ in maintaining CIN, we still could not ignore the possibility of other phosphorylation sites of KIF14 was phosphorylated by BUB1, since the phosphorylation band still exist in the KIF14^ΔSer1292^ mutant group in our Phos‐tag analyses, which was directly displayed in the in vitro kinase experiment. Hence, studying the phosphorylation of other sites in the future may be more beneficial for the completion of our work. In addition, it was worth noting that both BUB1 knockdown and inhibition downregulated the expression of KIF14. It is similar to previous findings that BUB1 directly phosphorylated CDC20 in vitro and catalysed the inhibition of the activity of APC/C (CDC20) ubiquitin ligase.^78^ Therefore, the phosphorylation of KIF14 by BUB1 not only activated the CIN signalling but was also likely to avoid its degradation by ubiquitination.

Taken together, our studies confirmed an essential role for BUB1 in promoting the growth and metastasis of ATC tumours, which were attributed to the induction of CIN through interacting with KIF14. These insights theoretically provide a valuable basis for the mechanisms of ATC occurrence and progression and final potential therapeutic target identification.

## AUTHOR CONTRIBUTIONS

**Tiefeng Jin:** Data curation (lead); writing – original draft (lead); writing – review and editing (lead). **Lingling Ding:** Data curation (equal); methodology (equal). **Jinming Chen:** Data curation (equal); formal analysis (equal); writing – original draft (equal). **Xiaozhou Zou:** Conceptualization (equal); resources (equal); software (equal). **Tong Xu:** Project administration (equal); supervision (equal); visualization (equal). **Zixue Xuan:** Investigation (equal); visualization (equal). **Shanshan Wang:** Data curation (equal); formal analysis (equal); software (equal); supervision (equal); writing – review and editing (equal). **Jianqiang Chen:** Formal analysis (equal); methodology (equal); supervision (equal); visualization (equal). **Wei Wang:** Investigation (equal); methodology (equal); project administration (equal); validation (lead); visualization (equal). **Chaozhuang Zhu:** Methodology (equal); resources (equal). **Yiwen Zhang:** Data curation (equal); software (equal). **Ping Huang:** Formal analysis (equal); supervision (equal); validation (equal). **Zongfu Pan:** Formal analysis (equal); supervision (equal); writing – original draft (equal); writing – review and editing (lead). **Minghua Ge:** Funding acquisition (lead); writing – original draft (lead); writing – review and editing (lead).

## FUNDING INFORMATION

Our research was supported by the National Natural Science Foundation of China under Grant (Nos. U20A20382, 82173157, 82273287), Natural Science Foundation of Zhejiang Provincial under Grant No. LY22H160041, Chinese Medicine Research Program of Zhejiang Province (Nos. 2022ZZ001 and 2021ZA006) and Zhejiang Medical and Health Science and Technology Project (Nos. 2022KY042 and 2021KY056). Zhejiang Provincial Program for the Cultivation of High‐level Health Talents (to Zongfu Pan).

## CONFLICT OF INTEREST STATEMENT

The authors have no conflict of interest.

## Supporting information


Figure S1

Data S1.

## Data Availability

The data that support the findings of this study are available on request from the corresponding author. The data are not publicly available due to privacy or ethical restrictions.
