# Supplementary figures and images for: BUB1/KIF14 complex promotes anaplastic thyroid carcinoma progression by inducing chromosome instability
Source: J Cell Mol Med. 2024 Mar 18;28(7):e18182. doi: 10.1111/jcmm.18182 (PMC10948175; doi:10.1111/jcmm.18182)

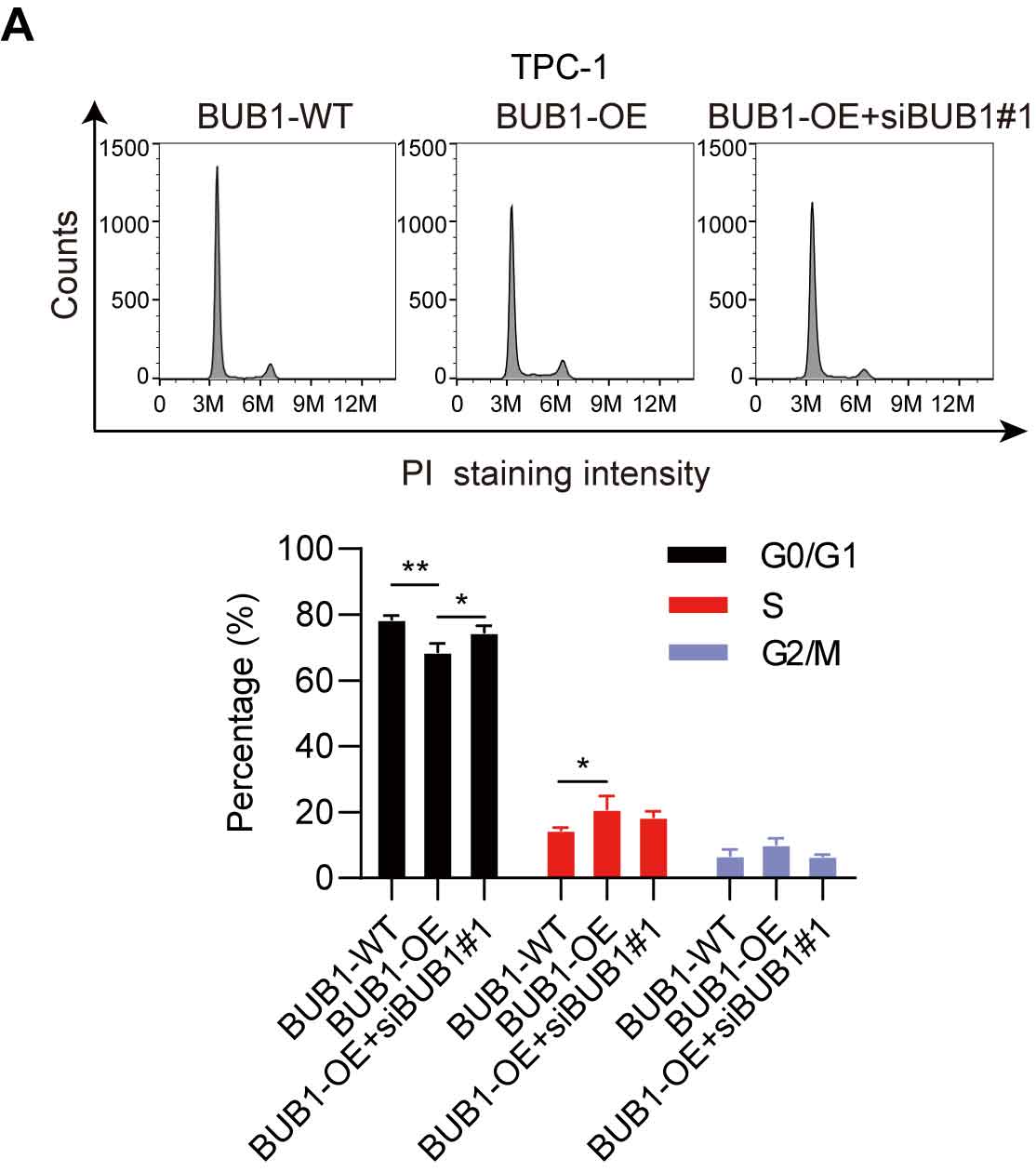

Supplement: Supplementary file 1 — Figure S1 [file JCMM-28-e18182-s001.jpg]
