# Supplementary material for: BUB1/KIF14 complex promotes anaplastic thyroid carcinoma progression by inducing chromosome instability
Source: J Cell Mol Med. 2024 Mar 18;28(7):e18182. doi: 10.1111/jcmm.18182 (PMC10948175; doi:10.1111/jcmm.18182)
Supplement: Supplementary file 2 — Data S1. [file JCMM-28-e18182-s002.docx]

Fig. s1. Overexpression of BUB1 promoted the cell cycle progression of papillary thyroid cancer cells. (A) Overexpression of BUB1 decreased the proportion of the G0/G1 phase in TPC-1 cells.
